# Three-dimensional construction of micrometer level in rat stomach by synchrotron radiation

**DOI:** 10.1186/s12938-021-00866-8

**Published:** 2021-03-20

**Authors:** Qiang Tao, Chenchen Gao, Xuehong Tong, Shizhen Yuan, Jingdong Xu

**Affiliations:** 1 School of Biomedical Engineering, Capital Medical University, Beijing, 100069 China; 2 Department of Physiology and Pathophysiology, School of Basic Medicine, Capital Medical University, Beijing, 100069 China; 3 Experimental Centre for Basic Medical Teaching, School of Basic Medicine, Capital Medical University, Beijing, 100069 China

**Keywords:** Synchrotron radiation phase-contrast imaging, 3-dimensional gastric structure images, Different stages

## Abstract

**Background:**

The structural changes of gastric mucosa are considered as an important window of early gastric lesions. This article shows an imaging method of the stomach that does not use imaging agents. X-ray phase-contrast images of different stages of gastric development were taken using micrometer level X-ray in-line phase-contrast imaging (XILPCI) technique on synchrotron radiation facility. The aim of the study was to demonstrate that the imaging technique is an appropriate method for micron imaging of the gastric structures.

**Methods:**

The stomachs of 4-, 6- and 12-week-old rats were removed and cleaned. XILPCI has 1000 times greater soft tissue contrast than that of X-ray traditional absorption radiography. The projection images of the rats stomachs were recorded by an XILPCI charge coupled device (CCD) at 9-μm image resolution.

**Results:**

The X-ray in-line phase-contrast images of the different stages of rats’ gastric specimens clearly showed the gastric architectures and the details of the gastro-duodenal region. 3-dimensional (3D) stomach anatomical structure images were reconstruction.

**Conclusion:**

The reconstructed gastric 3D images can clearly display the internal structure of the stomach. XILPCI may be a useful method for medical research in the future.

**Supplementary Information:**

The online version contains supplementary material available at 10.1186/s12938-021-00866-8.

## Background

The stomach is an important organ in the alimentary tract. However, the imaging observation of its development has not been realized yet. This mainly employs gastroscopy which could obtain the image at a millimeter level. There are high-resolution images of X-ray absorption of the human skeleton, but the images of the human abdominal organs remain poor. X-ray in-line phase-contrast imaging (XILPCI), using the X-ray phase-change after an X-ray passes through objects, has emerged as an imaging method and used to acquire micrometer level images of soft tissues.

XILPCI can be combined with computed tomography (CT) and form the phase-contrast CT. It is also a kind of the diffraction CT and will probably be an imaging method for soft tissues without using imaging agents. The micrometer level image resolution of XILPCI of soft tissues can reach 0.37 μm which make it able to obtain the more accuracy gastric structure. With the advantages mentioned above, this type of XILPCI method could be a good research method in the future of medicine.

A growing number of studies have shown that intestinal development has obvious temporal characteristics and is regulated by many factors [1]. The rapid growth and functional maturation of the stomach and small intestine in newborns may reflect an adaptation process, which include being exposed to an open environment and processing nutrient soon after the birth of newborns. Morphometrical analysis revealed that the growth rate is greater in the gastric corpus region than in the cardiac and pyloric regions, and the same increase is observed in the mucosal layer compared with the one in other layers. It has been shown that gastric mucosal cell proliferation was elevated during early postnatal development in the rat model [2]. Some evidence indicate that growth of gut mucosal tissues is associated with an enhanced DNA synthesis rate [3] and a decreased cell turnover rate in neonatal animals [4]. There are lots of researches on gastrointestinal cells, but few studies focus on the characteristic morphological in stomach and intestine of the rats. Our team focused on the XILPCI images of morphology and structure of normal rat stomach. Hence, we choose to use the young and adult rats as the objects of study.

## Results

### Rats’ gastric specimen characteristics in XILPCI projection images

**Fig. 1:**
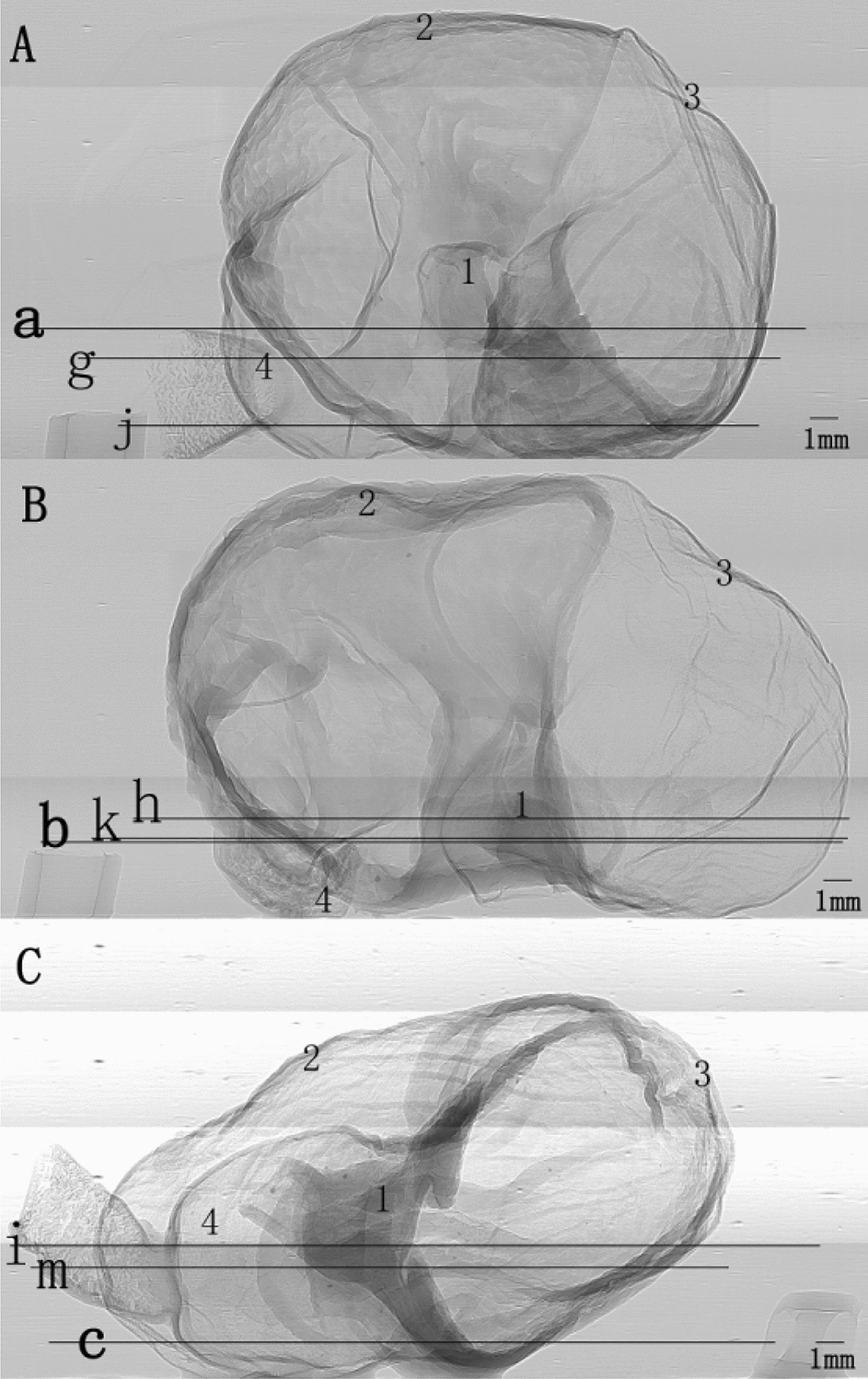
XILPCI projection images of rats’ gastric specimens. **A** A 4-week-old specimen showing a smooth gastric corpus wall. **B** A 6-week-old specimen showing the uneven gastric walls inside the stomach. **C** A 12-week-old specimen showing thick gastric corpus walls and more gastric corpus wrinkles. 1 Cardia, 2 Gastric Corpus, 3 Gastric Fundus, 4 Pylorus

**Fig. 2:**
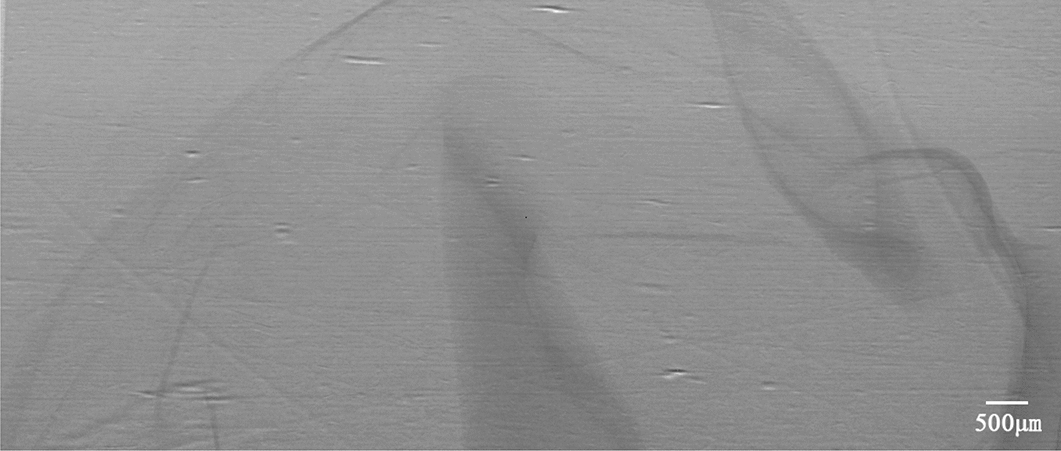
An X-ray absorption image of a rat stomach

To intuitively understand the changes of gastric morphology and structure in different age groups, the XILPCI projection images of gastric specimens are shown in Fig. 1. Changes were observed at different week in rats. Figure 1A shows the ordered and regular tissues of a 4-week-old young gastric normal specimen, and the gastric walls are smooth without any hyperplasia. The XILPCI image are more detailed than the X-ray traditional image of a normal gastric specimen [5–8], as we could only see the overlapping walls of the stomach in the absorption images, and the images are very fuzzy with an unclear internal texture (as shown in Fig. 2).

On XILPCI images, a 4-week-old image shows a uniform grey level, which indicates that the wall of the 4-week-old gastric corpus is as thick as the gastric fundus. It is obvious that the wrinkles of the fundus are abundant, while the gastric fundus is much thinner. In the middle of Fig. 1B, C, there is a demarcation line between the gastric corpus and the gastric fundus. Figure 1B demonstrates the characteristics of a 6-week-old adult gastric normal specimen XILPCI image. The gastric wrinkles are more extensive, but the wrinkles of the 6-week-old gastric fundus are fewer than those of the 4-week-old gastric fundus, as shown in the image that the grey of a 6-week-old gastric fundus is lighter than that of a 4-week-old fundus. The 6-week-old gastric corpus is thicker than the 4-week-old gastric corpus for the grey of a 6-week-old gastric corpus is deeper in image than that of 4-week-old. Figure 1C demonstrates the characteristics of mature gastric normal tissues from a 12-week-old normal gastric specimen. The wrinkles in the walls of the gastric corpus and duodenum are the most abundant.

## CT images of the same rats’ gastric specimens

**Fig. 3:**
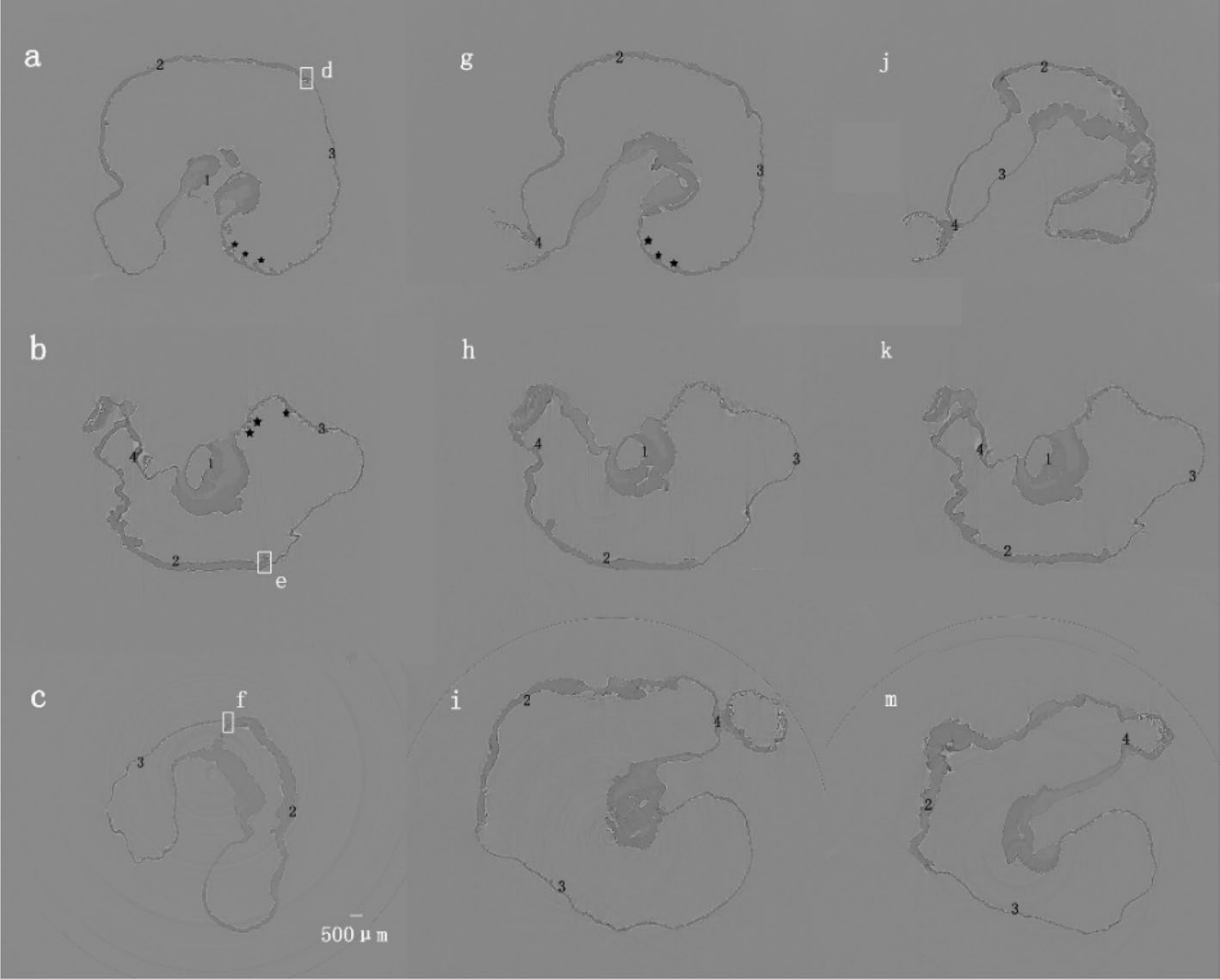
The XILPCI transverse CT images of the rats’ gastric specimens in Fig. 1. **a** The transverse images along the black line a, g and j are shown in Fig. 1. There are transverse CT images of a 4-week-old rat stomach. It can clearly show that there are grey changes in different gastric structures. The thickness of the gastric fundus and corpus are the same as the XILPCI image shows equal width of gastric wall, and it is obvious that there are multiple bulges in the fundus of the 4-week-old rat stomach. **b** The transverse images along the black line b, h and k as shown in Fig. 1. The XILPCI images show the wider gastric corpus than the fundus but thicker walls of gastric corpus than that of the fundus. This obviously shows that there are grey changes in the different gastric structures and the duodenum. The bulges have decreased in the fundus of the 6-week-old rat stomach. **c** The transverse images along the black line c, i and m as shown in Fig. 1. It can obviously be seen that there are many gastric corpus wrinkles in the 12-week-old gastric specimen. **★★★** represents the wrinkles of the gastric fundus. 1 Cardia, 2 Gastric Corpus, 3 Gastric Fundus, 4 Pylorus

The XILPCI 3-dimensional slices were rebuilt by means of a filter back projective algorithm. As shown in Fig. 3, the details can be visualized inside the gastric tissue from under the gross anatomy and the gastric inner surfaces clearly presented longitudinal branching wrinkle and gastric pits.

**Fig. 4:**
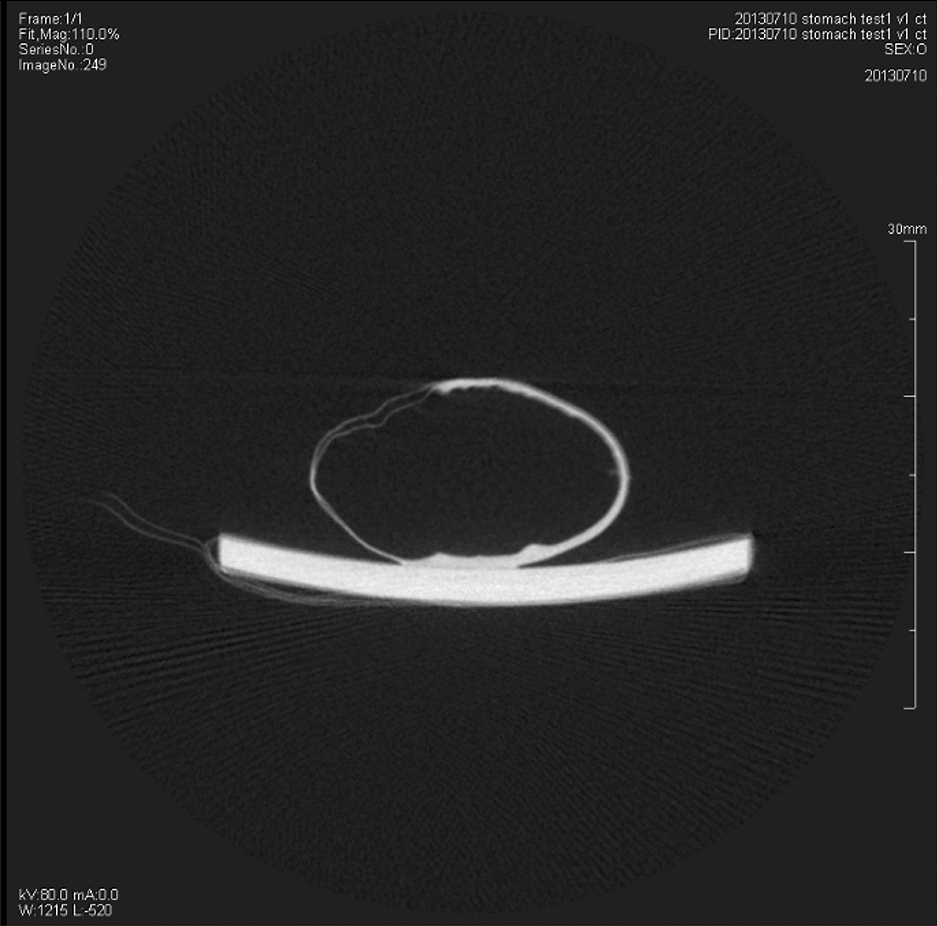
The traditional absorption CT image of the normal rat’s gastric specimen

Traditional absorption CT images of a normal gastric specimen were obtained by SIEMENS Inveon Scanners with minimum resolution of 11 μm and Inveon Acquisition Workplace with 1.5 Service Pack. The X-ray energy was 80 keV and 400 μA. The absorption CT image of a gastric specimen was Fig. 4. However, it was not clearer than gastric phase-contrast images and the wall fold of the normal gastric specimen cannot be observed.

**Fig. 5:**
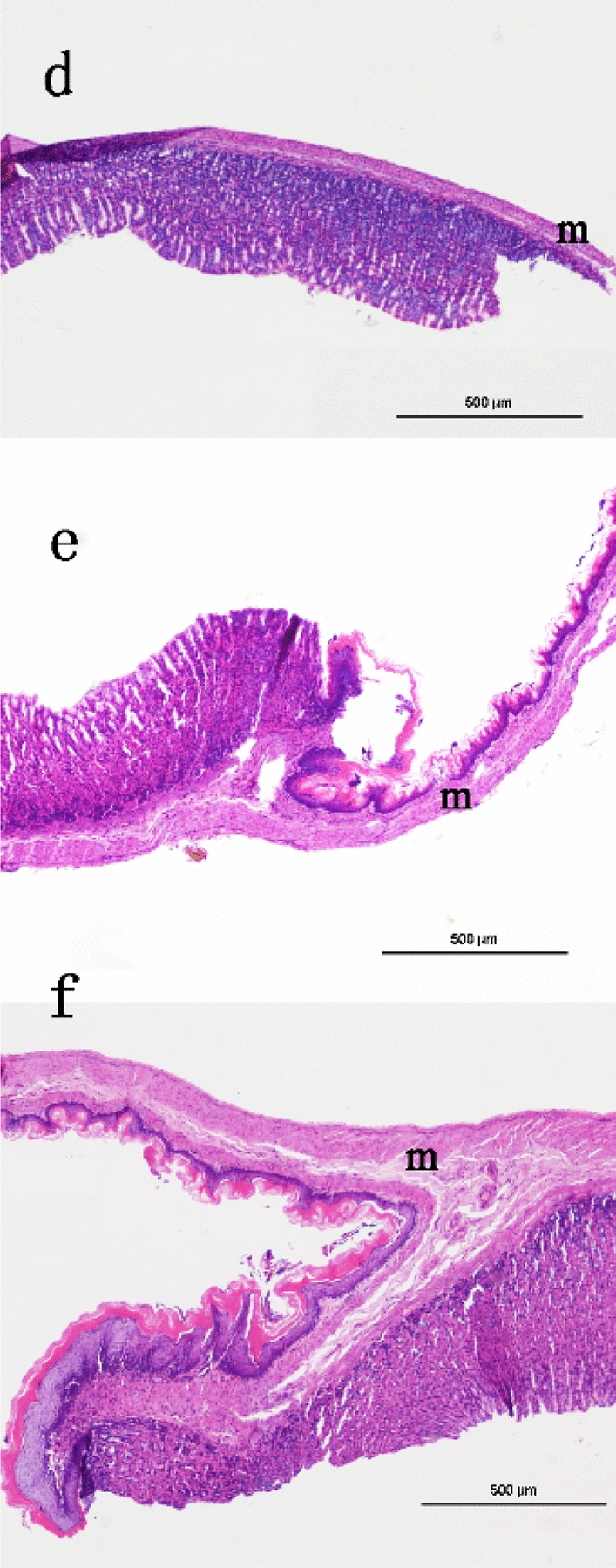
Rats’ gastric HE staining images. **d** A 4-week-old gastric specimen; **e** a 6-week-old specimen; **f** a 12-week-old gastric specimen

The d, e and f parts of the gastric specimens in Fig. 3 were taken out and fixed in formalin solution. Further analysis of these specimens show the same shape as Fig. 5, but XILPCI images could not show the clear internal structure the same as Fig. 5. At present, the gold standard for diagnosis is still biopsy. Hematoxylin–eosin (HE) staining processes are as follow: these gastric specimens were dehydrated and dried, and then the d, e and f part of specimens were embedded in paraffin. Paraffin-embedded sections were made into pathological section with 5 μm thickness and stained with HE to evaluate general morphology. In the body portion of the stomach after HE staining, the micrograph and macrograph showed a cross-section of the gastric wall (Fig. 5). Similar to the other parts of the gastrointestinal tract, there are four layers of structure in the gastric wall, which are outer mucosa, inner submucosa, muscular external layer, and serosa. During the growth and development of the rat, the fundus, formed by the upper curvature of the organ from the muscular external layer, is the thickest part at all three ages (Fig. 5d–f). There is an obvious line between the gastric fundus and corpus. A mucosa layer is also palpable, but with overt differences seen on HE staining, and we could see that the mucosa layer in the 4-week-old rats show more alkalinity than that in the 6- and 12-week-old rats, especially at 12 weeks (Fig. 5f). The line could also be observed in Fig. 5d–f between the gastric fundus and corpus.

## Three-dimensional reconstruction images of the same rats’ gastric specimens

**Fig. 6:**
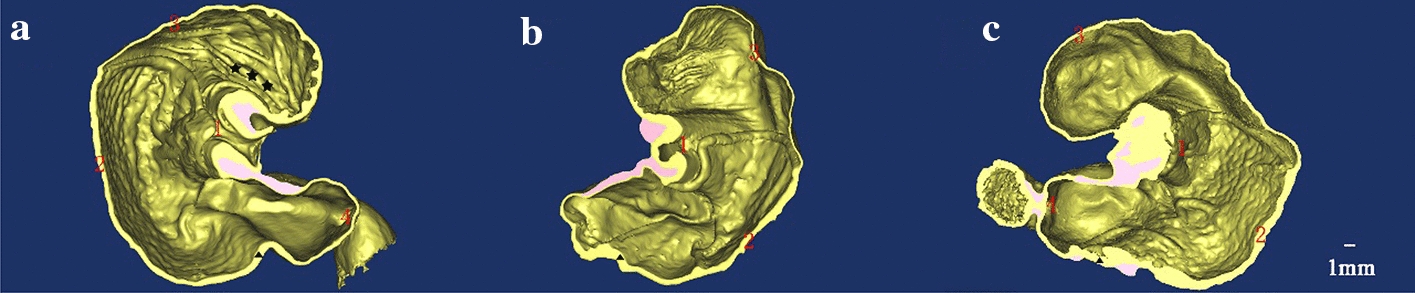
3D anatomical structure images of the coronal plane of different stages of the stomach. **a** 3D internal structure image of the 4-week-old rat stomach. The stomach is round with obvious wrinkles in the fundus part, and there is a clear demarcation line between the gastric fundus and gastric corpus. **b** 3-dimensional internal structure image of the 6-week-old rat stomach. The wrinkles of the fundus part are decreasing, and the gastric corpus is becoming rugged. **c** 3-dimensional internal structure image of the 12-week-old rat stomach. The stomach is elliptic, and the fundus part of it has become smooth. ★★★ represents the wrinkles of the gastric fundus. ▲ is the same size in the ** a**,** b** and **c** part of Fig. 6 and represents the thickness of the wall of the gastric corpus. 1 Cardia, 2 Gastric Corpus, 3 Gastric Fundus, 4 Pylorus

**Fig. 7:**
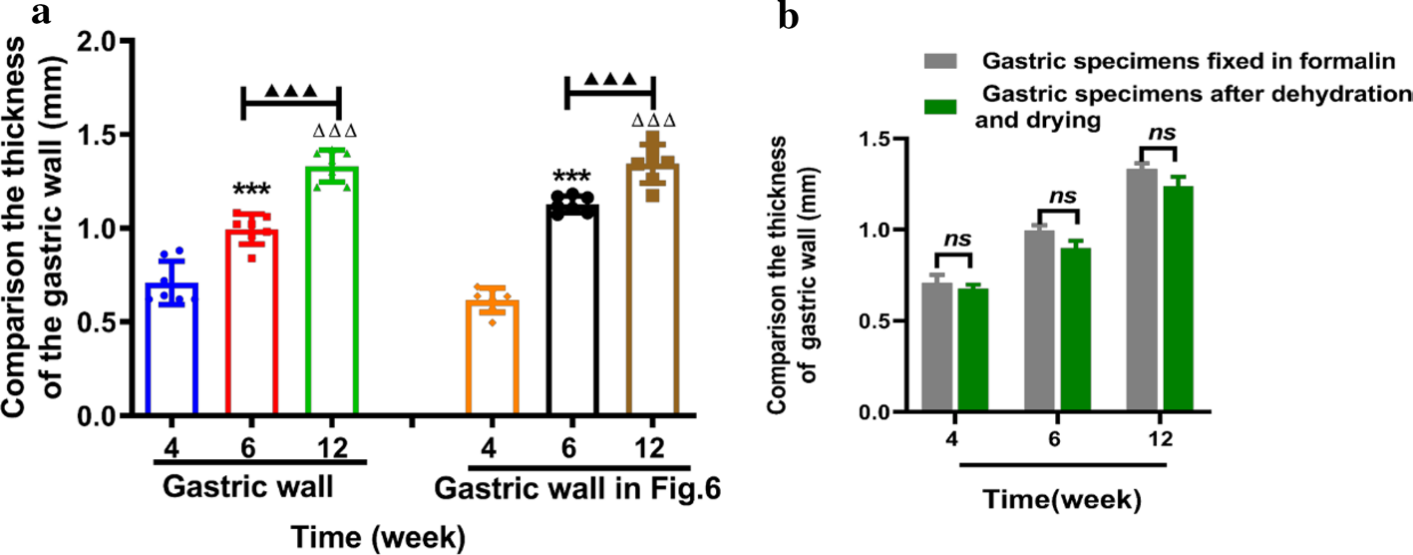
The comparison results of the thickness of the gastric wall at various stages between gastric specimens and 3D figures in Fig. 6 and gastric specimens after dehydration and drying. **a **The thickness of the gastric wall of the specimens is significant increased from 0.71 ± 0.04 mm to 0.99 ± 0.05 mm, 1.33 ± 0.32 mm about 28.28% (*p* < 0.001, *n* = 7), 46.61% (*p* < 0.001, *n* = 7) in the 4 weeks, 6 weeks and 12 weeks postnatal, respectively. And the thickness of gastric walls was calculated as shown in Fig. 6, with a marked enhancement from 0.62 ± 0.024 mm to 1.13 ± 0.02 mm, 1.34 ± 0.04 mm about 45.13% (* p *< 0.001, *n* = 7) and 53.73% (*p* < 0.001, *n* = 7). All the data at the same stage have no difference. Data represent mean ± S.E.M. (^***^P < 0.001, *P* < 0.001, ^▲▲▲^*P* < 0.001). ^***^4-week-old vs 6-week-old, 4-week-old vs 12-week-old, ^▲▲▲^6-week-old vs 12-week-old. **b** The thickness of gastric wall tends to decrease from 0.71 ± 0.044 to 0.62 ± 0.05 mm by 4.6% (*p* > 0.05, *n* = 7) in the 4 weeks models, from 1.00 ± 0.05 to 0.90 ± 0.04 mm by 9.6% (*p*>0.05, *n* = 7) in the 6 weeks models, and from 1.33 ± 0.08 to 1.210 ± 0.06 mm by 9.12% (*p*> 0.05, *n* = 7) in the 12 weeks models, respectively. These results showed that the gastric shape and structure unchanged, and the thickness of gastric wall decreased proportionally less than 10% within the credible range of no difference

To further reconfirm the advantage of the XILPCI 3-dimensional images, we reconstructed 3-dimensional images of the different stages of the stomach as shown in Fig. 6. The XILPCI 3-dimensional images show a clear structure of the interior stomach (showed as Additional file 1: Video S1), and we could see the clear images of the villus of the fundus in the young rat stomach. There is a clear demarcation line between the gastric fundus and corpus. The wall of the 12-week-old rat stomach is thicker than those of the 4-week-old. The conclusion here shows that XILPCI has a high anatomical accuracy for stomachs images. In addition, the thickness of the gastric wall at various stages was measured in Fig. 6 to compare the thickness of gastric specimens. As shown in Fig. 7, the results indicate that the average value of gastric wall at the same stage is very similar, and the measurement of gastric wall by 3-dimensional images in Fig. 6 has certain reference significance. Just as shown in Fig. 7a, the thickness of 4-week-old gastric wall for the right panel is lower than that of the left one, but the thickness of 6-week-old gastric wall is higher for the right panel, probably because of the randomness of specimen selection and the existence of measurement error. To further confirm the experimental evidence, we measured the thickness of gastric wall of the dried specimens which can be directly used for XILPCI experiments. As the Fig. 7b shown, it is clearly indicated the different types of gastric specimens shrink proportionally. However, these results are also in line with the requirements of statistics and within a reasonable range.

## Discussion

Limitations of XILPCI must be overcome if XILPCI is to have wider applications. One limitation is that it takes around 1 h to shoot a larger specimen as it requires images shooting in several segments. The experiment is only effective for static specimens, so the device must be improved to shorten the imaging time required for living specimens. In addition, movement artifacts will appear when the specimens being taken are moving. In this experiment, because of the high resolution of the imaging, a merely small movement can produce imaging artifacts. Specimens shrinkage occurs when the specimens are taken on the sample table, which also produces movement artifacts under X-ray irradiation. The current solution is to make the specimen as dry as possible so as to shorten the experiment time. Movement artifacts can also affect the image quality and require further solution in later researches.

The CCD camera of BL13W1 of SSRF can obtain a resolution of 0.37 μm, making the XILPCI helpful for angiography. At present, Applications on humans in the field of phase-contrast mammography have been reported using both synchrotron radiation and conventional sources [9]. With further development, XILPCI would be a valuable imaging method for medical researches.

## Conclusion

In summary, we have applied the XILPCI method to imaging of the rat stomach without the need for imaging agents. The XILPCI projection images showed that the development of the normal gastric structure may cause thickening of the gastric wall and coarseness of the gastric texture.

XILPCI is an imaging method using the X-ray phase variation. This makes it able to achieve micron-scale image resolution of biological tissue and can probably replace the traditional X-ray absorption method with synchrotron radiation sources. Due to the advantages mentioned above, the XILPCI method is likely to be widely used in the future at a low cost.

## Methods

### Setup and specimens

**Fig. 8:**
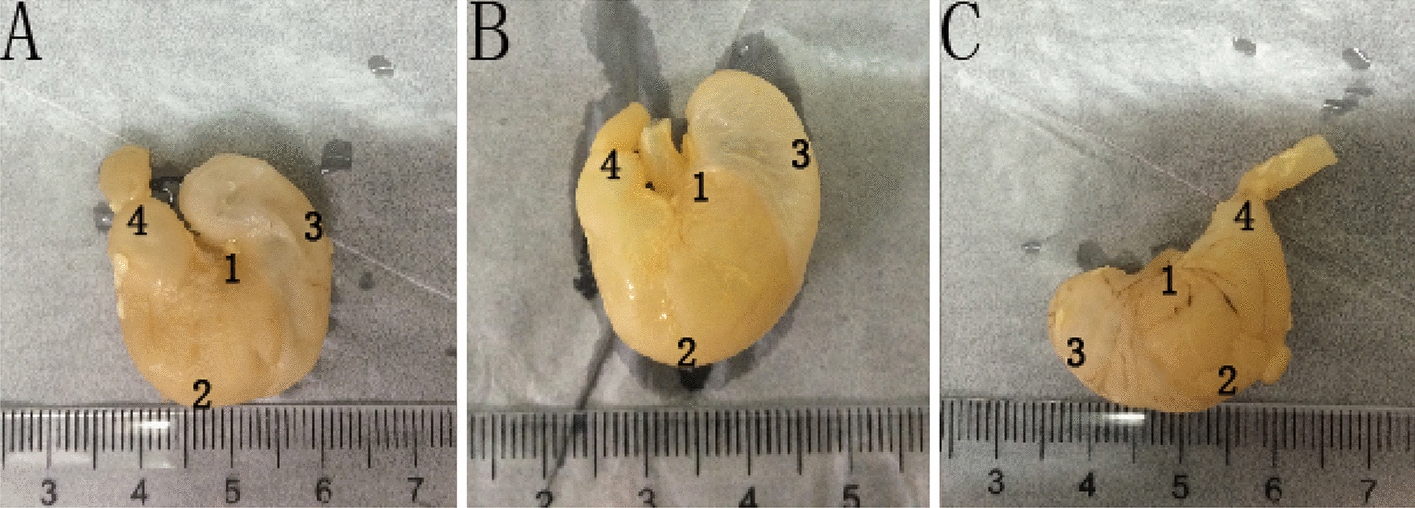
Rats’ gastric specimens. **A** A 4-week-old gastric specimen. **B** A 6-week-old gastric specimen. **C** A 12-week-old gastric specimen. 1 Cardia, 2 Gastric Corpus, 3 Gastric Fundus, 4 Pylorus. All the positions have been noted on the gross anatomy

Twenty-one Sprague–Dawley healthy male rats were purchased from the Animal Centre of Capital Medical University in China. The rats were randomly divided into three groups with seven rats in each one. The rats were housed in cages under a controlled temperature of 22.0 ± 1.0 °C and 12-h light–dark cycles. They were fed on standard laboratory chow and water and allowed to acclimate for more than 7 days. The rats were starved for 12 h and killed under anesthesia with an intraperitoneal injection of pentobarbital sodium at 4, 6 and 12 weeks of age. The stomach of the rat was removed and the stomach cavity is filled with 10% formalin to maintain the morphological structure of the stomach for X-ray image scanning. Stomach specimens were shown Fig. 8. These stomach tissues were cleaned with normal saline, fixed in 10% neutral phosphate-buffered formalin solution for 24 h and embedded in paraffin. 5 μm thick of the specimens were obtained from the stomach and stained with HE staining. This animal study was in strict accordance with the recommendations in the Guide for the Care and Use of Laboratory Animals of the National Institutes of Health. The animal welfare Committee on the Ethics of Animal Experiments of Capital Medical University (Protocol No. AEEI-2016–079) approved the experiment protocol. All surgeries were performed under pentobarbital sodium anesthesia, and all efforts were made to minimize suffering.

## Imaging principle of XILPCI

XILPCI experiments were done at synchrotron radiation [10] facility. Synchrotron radiation is electromagnetic radiation which is emitted by relativistic charged particles traveling along a turning orbit under the action of electromagnetic field. Synchrotron radiation, as a light source, has an obvious high-brilliance and flux, wide energy spectrum and very short pulses.

**Fig. 9:**
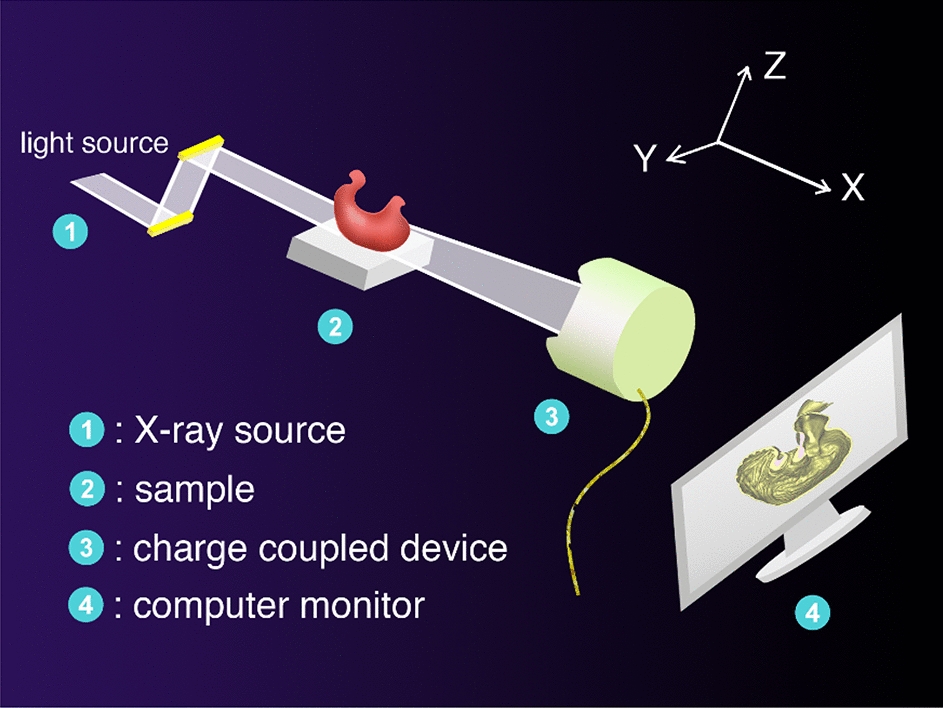
Schematic diagram of BL13W1 beam line of SSRF. ① The light source is used for the calibration location of the light, specimen and the CCD. ② A multidimensional sample table. The specimens are placed on the sample table to rotate and their images are then obtained at different angles. ③ An X-ray CCD. It obtains specimens’ projection images with high-resolution. ④ Data processor. It could calculate optical density from the CCD to the specimens and image conversion

The gastric XILPCI experiments were performed using the BL13W1 beamline of the Shanghai Synchrotron Radiation Facility (SSRF). The BL13W1 beamline partial facility of SSRF was depicted as shown in Fig. 9. A. Snigirev [5, 11–13] obtained phase-contrast images using a synchrotron radiation light in 1995. The XILPCI method can use multi-color light sources, therefore, eliminate the need for the burdensome complexity of a monochrome system.

The complex refractive index n can be used to depict the XILPCI characteristics. The refractive index n is smaller than the number 1 and n formula is shown as follows:

1 $$n = 1 - \delta - i\beta .$$

After X-rays go through an object, their phase and amplitude change. Real component presents the phase changes, while imaginary part presents the amplitude attenuation. In XILPCI of lighter elements (C, H, O, etc.) of the object, δ is 1000 times greater than β, so the phase-change quantity is much larger than the change quantity of X-ray absorption attenuation. There is a phase-contrast imaging of micron-scale image resolution, so XILPCI images can show microstructures of objects.

## Steps of XILPCI

Specific experimental methods: first, to reduce the XILPCI artifacts caused by specimen deformation, the specimens are placed in the air and dried for a period of time. Then the dry specimens were wrapped with insulating materials and placed on the sample table.

After evaluating different levels of X-ray energy, we chose the energy level of 17.5 keV to be used for this experiment. The energy value can be changed and is determined according to the specimen size. Different specimens have different energy values to obtain clear X-ray phase-contrast images. The images were whiter if the energy was higher than 17.5 keV, and the imaging exposure time also increased when the energy was lower than 17.5 keV. Images will be dark if the exposure time is not appropriate. It will also take a longer time to shoot those images required for CT if the exposure time increases and gastric specimens will undergo serious deformation. Therefore, 17.5 keV is an optimal parameter of comprehensive factors. The distance from the light source to the specimen was 59.3 m and the detector was 60 cm from the specimen to CCD, with 9-μm image resolution and an exposure time of 8 ms. It took more 20 min to obtain XILPCI projection images of a gastric specimen using 0.1 degree steps from 0 to 180 degrees over the gastric specimen.

## Gastric wall thickness measurement

To further compare the effects of stomach drying and atrophy caused by exposure to air under the test conditions on the structure, the specimens fixed with 10% formalin were exposed to air at the same temperature and for the same duration in this experiment. After 20 min of drying, the thickness of the gastric wall was measured under stereoscope with the vernier caliper. Three times for each position, seven arbitrary parts of gastric walls of all of dry specimens were selected and the average value was taken. The value is expressed as Mean ± SEM.

Pre-dehydration wall thickness measurement was the same conditions with the dehydration wall thickness. The stomach was taken out from 10% formalin solution and kept moist. The gastric walls thickness was measured at seven arbitrary parts of all of moist specimens, three times for each position, under the same stereoscope with the same vernier caliper. For further statistical significance, all gastric walls were randomly selected. The value is also expressed as Mean ± SEM.

## Histology and pathology scoring

Stomach tissue specimens were dewaxed and stained with the HE staining. Sections were fixed on microscope slides and observed with an Olympus DP72 MacroView (Japan). Histological scoring was based on a previously adapted scoring system.

## Statistical analysis

The data were analyzed using GraphPad Prism 5.0 software package (GraphPad Software Inc., San Diego, CA, USA) of variance of the gastric wall thickness and all values were expressed as mean and standard error of mean (S.E.M.); *n* was the number of animal in each experiment. The differences among groups were analyzed using a one-way analysis of variance followed by Dunnett’s multiple comparison. A *p* value less than 0.05 was considered statistically significant. The power of the results was 86.5% by the software power analysis and specimen size software.

## Supplementary Information

**Additional file 1: Video S1.** 3D video of a 4-week-old rat stomach.

## Data Availability

The datasets generated during the current study are available from the corresponding author on reasonable request.

## Abbreviations

XILPCI: X-ray in-line phase-contrast imaging
CCD: Charge coupled device
BL13W1: Imaging and biomedical application beamline
SSRF: Shanghai Synchrotron Radiation Facility
3D: Three dimensional
